# A Novel 26-bp Insertion in the *PpMYB10.1* Promoter Is Associated with Anthocyanin Deficiency in Peach (*Prunus persica*) Fruit Skin

**DOI:** 10.3390/ijms272110027

**Published:** 2026-11-09

**Authors:** Zhenhua Lu, Lei Pan, Zhenyu Huang, Liang Niu, Guochao Cui, Wenfang Zeng, Lifeng Liu, Suke Wang, Luwei Wang, Hao Wang, Zhiqiang Wang

**Affiliations:** 1 Zhengzhou Fruit Research Institute, Chinese Academy of Agricultural Sciences, Zhengzhou 450009, China; panlei@caas.cn (L.P.); niuliang@caas.cn (L.N.); cuiguochao@caas.cn (G.C.); zengwenfang@caas.cn (W.Z.); liulifeng@caas.cn (L.L.); wangsuke@caas.cn (S.W.); 15839552412@163.com (L.W.); haowang0110@126.com (H.W.); wangzhiqiang@caas.cn (Z.W.); 2 Key Laboratory of Genetic Resource Evaluation and Application of Horticultural Crops (Fruit), Ministry of Agriculture and Rural Affairs, Zhengzhou 450009, China; 3 Henan Joint International Research Laboratory of Fruit Tree Genomics, Zhengzhou 450009, China

**Keywords:** *Prunus persica*, fruit skin color, anthocyanin deficiency, promoter activity, marker-assisted selection

## Abstract

Fruit skin color is a decisive agronomic trait governing the commercial value of peaches; although *PpMYB10.1* is known to regulate anthocyanin accumulation in peach, the specific promoter structural variant that silences *PpMYB10.1* expression in non-red skin types has not been identified and functionally validated. Here, using a first backcross (BC1) population, we integrated bulked segregant analysis sequencing (BSA-seq), transcriptome profiling, and whole-genome resequencing to map the causal locus to a 453.083-kb interval on chromosome 3 harboring the R2R3-MYB transcription factor *PpMYB10.1*. Transient overexpression and virus-induced gene silencing (VIGS), together with stable tobacco transformation, confirmed that *PpMYB10.1* regulates the non-red skin phenotype in this population. Sequence comparison revealed a 26-bp insertion in the *PpMYB10.1* promoter of non-red fruit; reporter assays demonstrated that this insertion strongly reduces promoter activity and silences *PpMYB10.1* expression. A co-dominant InDel marker co-segregated with the skin color phenotype in all tested accessions and populations. Our findings suggest a promoter structural variant is associated with the molecular switch controlling anthocyanin accumulation in peach skin and provide a practical tool for marker-assisted selection.

## 1. Introduction

Higher plants display diverse colors (white, pink, red, purple, and blue) owing to different types and concentrations of anthocyanins [1,2]. Anthocyanins are a group of water-soluble compounds belonging to the flavonoid class of secondary metabolites [3]. Numerous studies have focused on the coloration mechanisms of leaves, petals, pericarps, and seed coats in horticultural plants [4,5,6,7]. Anthocyanin accumulation in plants is regulated and affected by light, temperature, nutritional conditions, and biotic and abiotic stresses [8,9,10]. Several genes in the anthocyanin pathway have been identified and characterized, including those encoding 4-coumaroyl-coenzyme A ligase, chalcone isomerase, flavonoid 3-hydroxylase, dihydroflavonol-4-reductase, anthocyanin synthase, and flavonoid-3-O-glucosyltransferase [6]. Among the genes involved in this pathway, MYB transcription factors are considered the primary determinants of anthocyanin synthesis in higher plants. The MYB protein belongs to a large family and exhibits diverse functional regulation of primary and secondary metabolism, especially phenylpropanoid metabolism, cell shape, hormone responses, and biotic and abiotic stresses [11,12]. MYB transcription factors are classified into four types based on the number of MYB domains: 1R-MYB, R2R3-MYB, 3R-MYB, and 4R-MYB [13,14,15]. R2R3-MYB proteins represent a major class of anthocyanin biosynthesis regulators in plants, while other MYB subfamilies also contribute to flavonoid pathway regulation in certain species [16,17]. Recent studies have shown that the regulation of anthocyanin biosynthesis genes occurs mainly through the MYB–bHLH–WD40 (MBW) complex, comprising MYB transcription factors, basic helix-loop-helix (bHLH) proteins, and WD40 repeat proteins, which activates anthocyanin and proanthocyanidin biosynthesis [14,15,18]. MYB transcription factors positively regulate the anthocyanin pathway in many fruit species, including peach (*Prunus persica* L.) [19,20,21], grapevine (*Vitis vinifera*) [22], apple (*Malus domestica*) [10,23], and pear (*Pyrus communis*) [24].

*P. persica* belongs to the Rosaceae family and is among the most important deciduous fruit trees, with China accounting for approximately 48% of global peach production area [25]. As the center of origin of peaches, China possesses rich germplasm resources with diverse root, stem, flower, fruit, and leaf coloration [6,21,26,27,28]. Fruit skin color is a key factor determining the commercial quality and value of peaches and is an essential quality trait for peach breeders. In Asia, especially in China, pure white or yellow peach fruits are produced by bagging to block anthocyanin accumulation, which meets consumer preferences but increases input and labor costs. Therefore, leveraging germplasm resources to breed cultivars with anthocyanin-deficient fruit skin is a crucial strategy for resolving this industrial problem.

Several studies have addressed the molecular mechanisms underlying anthocyanin accumulation in various plants. In the present study, we adopted forward genetic approaches to identify the genes responsible for anthocyanin deficiency in peach fruit skin. A BC1 segregation population was established to determine the genetic characteristics of this trait. Bulked segregant analysis combined with next-generation sequencing (BSA-seq) was used to map the gene locus. Through integrated RNA-seq, genome resequencing, transient gene overexpression, and gene silencing, we confirmed the function of the candidate gene *PpMYB10.1*, which causes the anthocyanin-deficient phenotype. The tissue specificity of the *PpMYB10.1* promoter was characterized, and a 26-bp insertion was found to disrupt promoter activity, hindering gene expression. For breeding purposes, we developed an InDel marker to distinguish between non-red and red skin phenotypes at an early stage. This study identified a key anthocyanin-regulating gene that specifically controls peach fruit skin color, providing a valuable tool for marker-assisted selection of desired fruit skin color types.

## 2. Results

### 2.1. Genetic Characteristics of the Fruit Skin Color Trait in Peach

**Figure 1:**
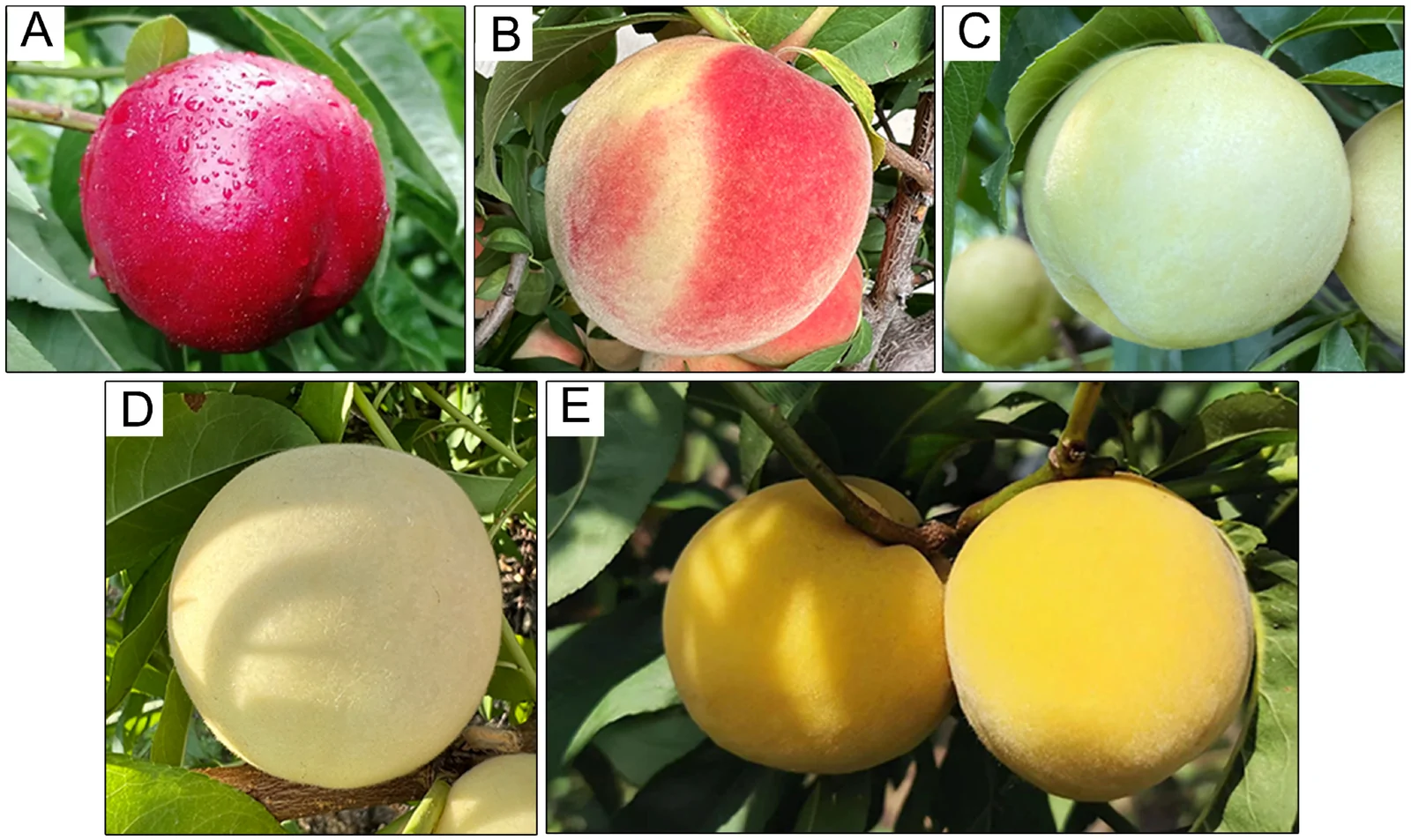
Phenotypes of the peach fruit skin color trait. (**A**) ‘03-33-7’. (**B**) ‘09-bei8-25’. (**C**) ‘Zhongtaobaiyu’. (**D**,**E**), Hybrids showing anthocyanin deficiency in fruit skin with white and yellow flesh, respectively.

Phenotypic analysis revealed that all F1 individuals from the ‘03-33-7’ × ‘Zhongtaobaiyu’ population (Figure 1A,C) showed partially red skin (Figure 1B). Of the 180 BC1 individuals generated from the cross ‘09-bei8-25’ × ‘Zhongtaobaiyu’, 88 showed red skin and 92 showed non-red skin, corresponding to a 1:1 Mendelian ratio (*χ*^2^ = 0.089, df = 1, *p* = 0.766). This indicated that the non-red skin type was controlled by a single recessive allele at the *PpMYB10.1* locus (denoted as r), while the red/partial red phenotype was conferred by the dominant allele (denoted as R). Specifically, ‘Zhongtaobaiyu’ (non-red skin) is homozygous recessive (rr), ‘09-bei8-25’ (partially red skin) is heterozygous (Rr), and the BC1 progeny segregate is 1 Rr (red/partial red): 1 rr (non-red). We observed that ‘Zhongtaobaiyu’ (Figure 1C) and half of its backcross progeny exhibited an anthocyanin-deficient phenotype specifically in the fruit skin (Figure 1D,E).

### 2.2. Anthocyanin Differences in the Fruit Skin of Red and Non-Red Peaches

**Figure 2:**
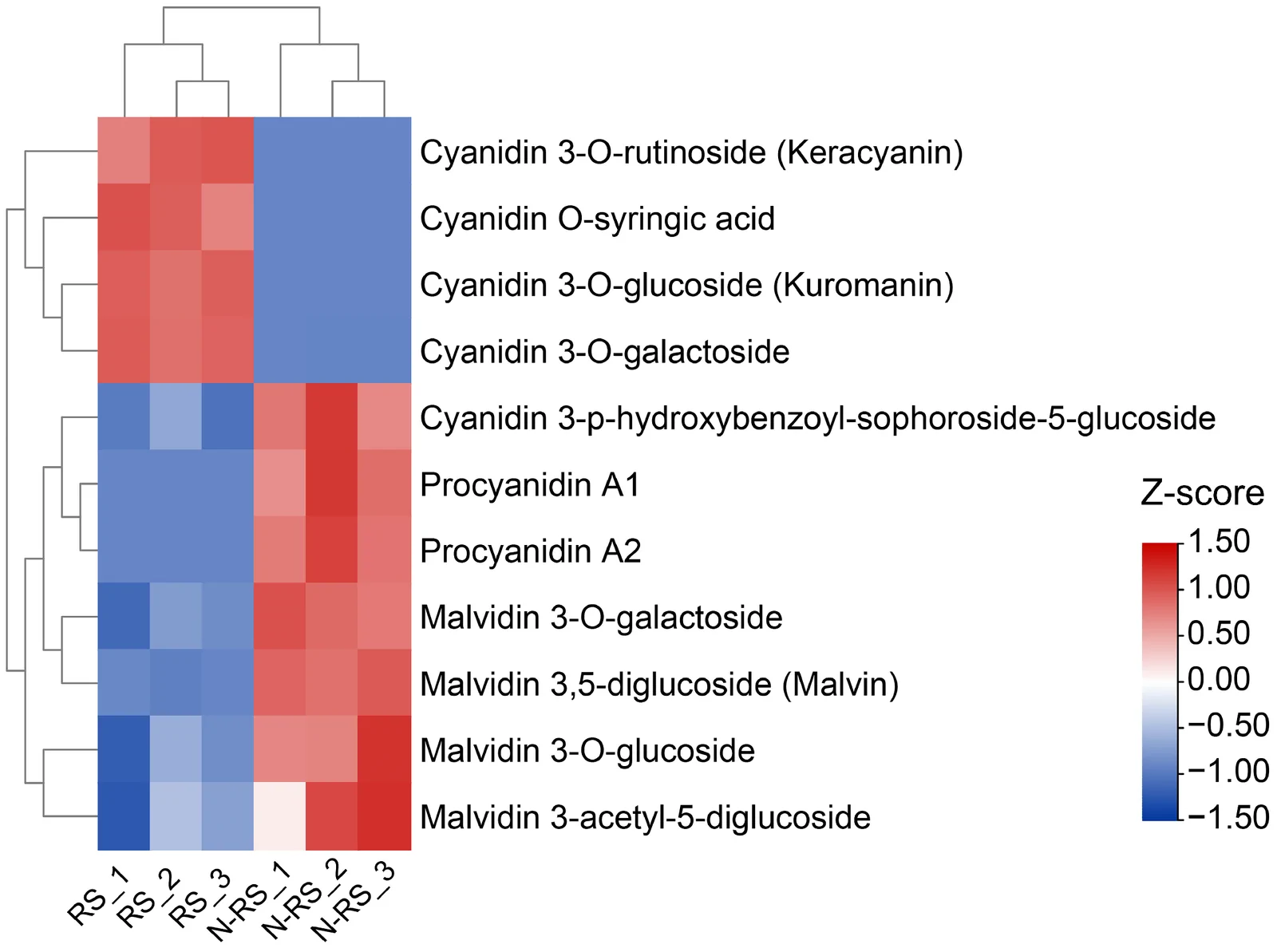
Heatmap of anthocyanin component profiles in red and non-red skin type peach fruits. RS, red skin type; N-RS, non-red skin type. Numbers following RS and N-RS indicate the three biological replicates. The color scale represents Z-score normalized relative abundances across all samples.

To explore the anthocyanin content in peach fruit skin, HPLC-MS/MS was used to detect differences between red and non-red skin types. In total, 11 major flavonoid components showed significant differences in content between the two types, comprising 9 anthocyanins and 2 proanthocyanidins. Of these, four anthocyanins (cyanidin-3-O-rutinoside, cyanidin O-syringic acid, cyanidin 3-O-glucoside, and cyanidin 3-O-galactoside) were more abundant in the red skin type than in the non-red skin type (Figure 2). Meanwhile, five anthocyanins (cyanidin 3-p-hydroxybenzoyl-sophoroside-5-glucoside, malvidin 3-O-galactoside, malvidin 3,5-diglucoside, malvidin 3-O-glucoside, and malvidin 3-acetyl-5-diglucoside) and two proanthocyanidins (procyanidin A1 and A2) were more abundant in the non-red skin than in the red skin fruit. Procyanidin A1 in the non-red skin type was approximately 3214-fold higher than that in the red skin type, indicating the accumulation of procyanidin A1 in non-red skin fruit. We hypothesize that this may reflect redirection of the flavonoid pathway flux toward proanthocyanidin biosynthesis when anthocyanin synthesis is suppressed.

### 2.3. Gene Mapping Using BSA-Seq Analysis with SNP and InDel Markers

**Figure 3:**
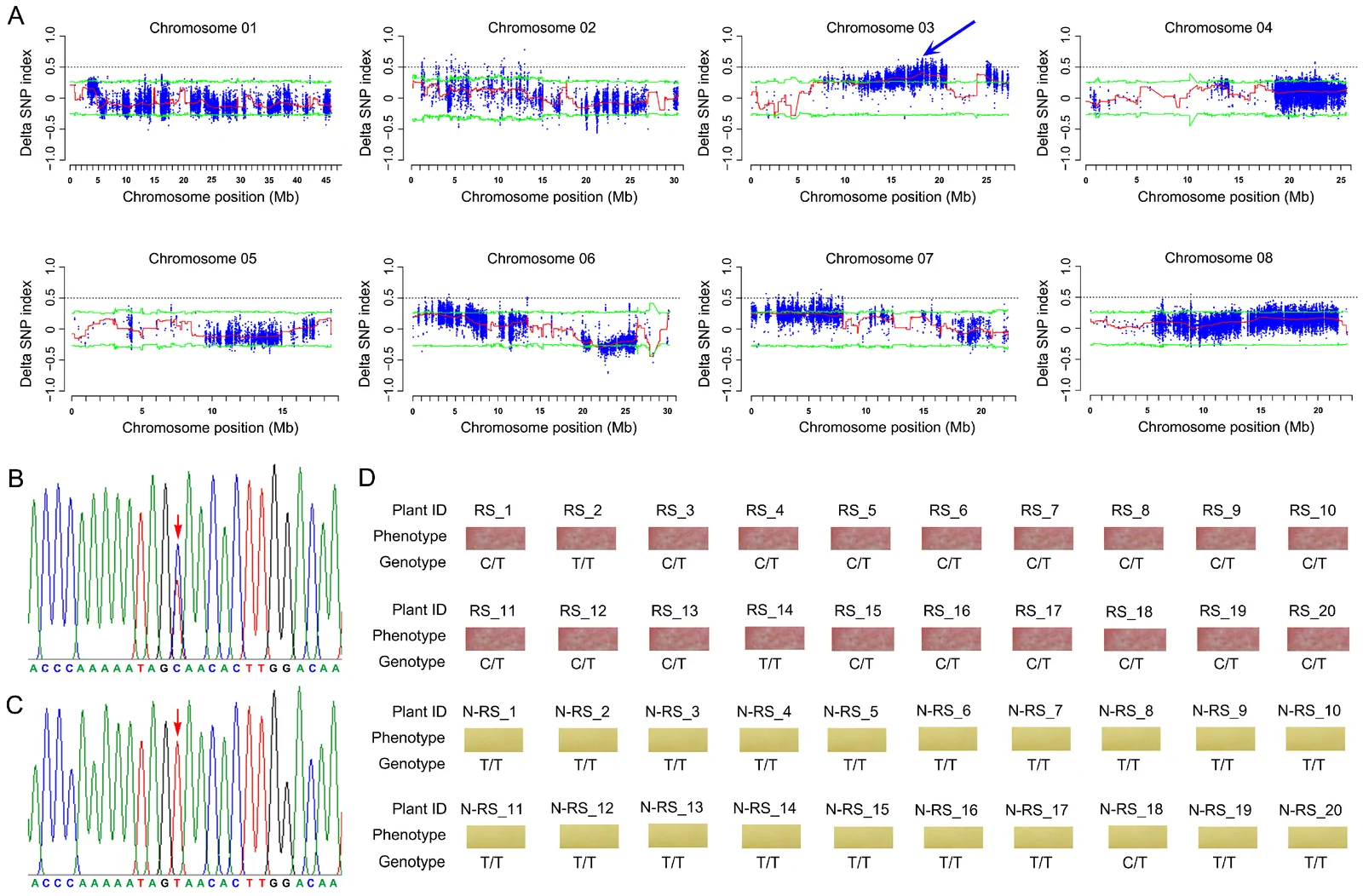
Genetic mapping and marker validation of the peach fruit skin color trait. (**A**) Distribution of Δ(SNP-index) on the eight peach chromosomes. The red line indicates the fitted values; the green line represents the 95% confidence interval; and the blue arrow indicates the candidate locus. (**B**,**C**) Sanger sequencing results of the two genotypes of SNP marker Pp03-18,854,098. The red arrow indicates the location of this SNP. (**D**) Genotyping results in 40 hybrid individuals. RS, red skin type; N-RS, non-red skin type.

Using BSA-seq analysis, the gene locus controlling fruit skin color was mapped based on 180 BC1 offspring. Whole-genome resequencing data of ‘09-bei8-25’ and ‘Zhongtaobaiyu’ at more than 60× depth was used to correct errors in the two gene pools. The raw data (Q20 ≥ 96.85%, Q30 ≥ 91.77%) comprised 28.26 Gb in the non-red skin pool and 23.24 Gb in the red skin pool. After removing the barcodes, the clean data were 28.24 and 23.22 Gb, respectively. A total of 181,002,328 reads with 38.75% GC content and 97.85% average depth in the non-red skin pool, and 148,759,655 reads with 38.81% GC content and 81.53% average depth in the red-type pool, were aligned against the peach reference genome v2.0. After filtering based on the data processing criteria (SNP-index < 0.3 in both pools, SNP depth < 7, and missing SNP-index in one pool), 106,106 SNPs and 69,050 InDels were generated for further analyses. After calculating the delta SNP-index, the candidate region between 17.409 and 19.635 Mb was located on chromosome 3, showing a peak of approximately 0.5 for the delta SNP-index value (Figure 3A). An SNP marker (Pp03-18,854,098) was designed and detected using Sanger sequencing to genotype 40 segregating individuals from the ‘09-bei8-25’ × ‘Zhongtaobaiyu’ population, revealing a tight linkage relationship between phenotypes and genotypes (Figure 3B–D).

### 2.4. Candidate Gene Identification Integrating Resequencing and RNA-Seq Data

**Figure 4:**
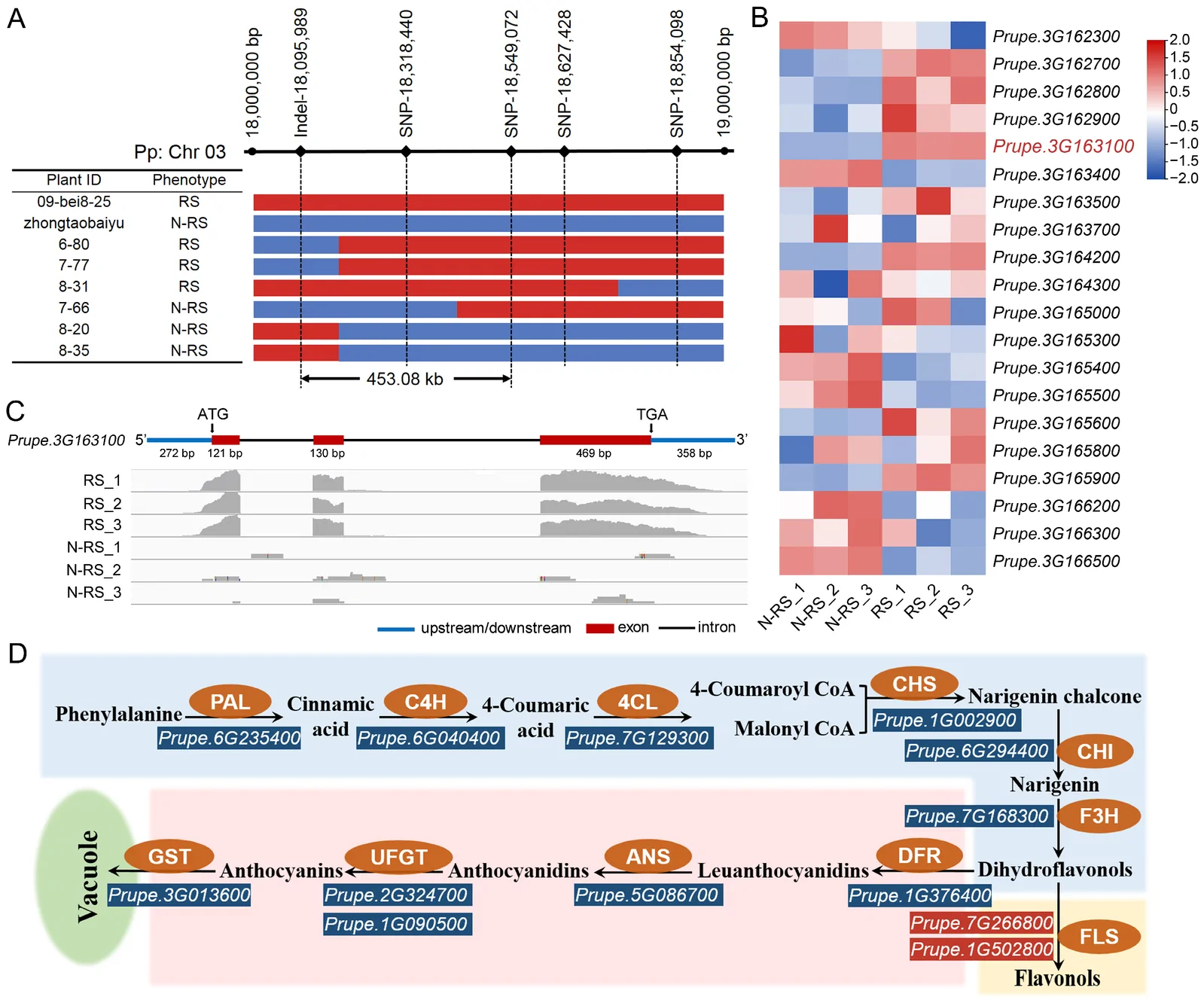
Candidate gene mining by integrating fine mapping and RNA-seq. (**A**) Fine-mapping results with six recombinant individuals. (**B**) Expression patterns of genes in the fine-mapping region. (**C**) RNA-seq results of *PpMYB10.1* viewed by IGV. (**D**) Expression patterns of anthocyanin pathway genes. Blue and red indicate down- and up-regulated genes in non-red vs. red skin, respectively. RS, red skin type; N-RS, non-red skin type.

SNP and simple sequence repeat (SSR) markers were designed in the genomic region with the highest linkage. These markers were used to analyze the genotypes of the 180 BC1 individuals to narrow the interval of the locus controlling peach skin color. BAM files were viewed using IGV software (version 2.19.8) to design SNP markers that were heterozygous in ‘09-bei8-25’ and homozygous in ‘Zhongtaobaiyu’. Six SNP and one SSR marker labeled with FAM were designed, spanning a region between 17.409 and 19.635 Mb (Table S1). After genotyping the 180 BC1 individuals, six informative recombinants were obtained, and the candidate gene was fine-mapped between the markers Pp03-InDel-18,095,989 and Pp03-SNP-18,549,072 with an interval length of 453.083 kb (Figure 4A, Table S2).

To identify the key genes controlling fruit skin color within the fine-mapping region, RNA-seq analysis was performed using fruit skin samples from non-red and red type peaches. For the six libraries, 51.99–56.61 million raw reads and 40.66–44.00 million clean reads were generated per sample, totaling approximately 38.4 Gb of clean data. Q20 and Q30 values were ≥93.9% and ≥84.9%, respectively, and clean-read ratios ranged from 77.4% to 80.6%. Twenty expressed genes were located within the mapped region, among which three differentially expressed genes, *Prupe.3G162800*, *Prupe.3G163100*, and *Prupe.3G164200*, showed |log2(fold change)| ≥ 1 between the two types of peach fruit skin (Figure 4B, Table S3). Remarkably, *Prupe.3G163100*, encoding the *PpMYB10.1* transcription factor, showed 97-fold higher expression in the fruit skin of the red type than in the non-red-type fruit (Figure 4C, Table S3).

Furthermore, we investigated the expression patterns of the key genes involved in the anthocyanin metabolism pathway. We identified ten genes that exhibited differential expression between the red and non-red fruit skin types (Figure 4D, Table S4). Among them, one flavonol synthase gene (*Prupe.7G266800*) was up-regulated in the non-red fruit skin type, whereas the other nine genes—including PAL (*Prupe.6G235400*), C4H (*Prupe.6G040400*), CHS (*Prupe.1G002900*), F3H (*Prupe.7G168300*), DFR (*Prupe.1G376400*), ANS (*Prupe.5G086700*), UFGT (*Prupe.1G090500* and *Prupe.2G324700*), and GST (*Prupe.3G013600*)—were down-regulated in the non-red fruit skin type. In particular, GST (*Prupe.3G013600*) was not expressed in the non-red skin type but was highly expressed in the red skin type, consistent with *PpMYB10.1* expression levels.

### 2.5. Functional Validation of PpMYB10.1 Using Overexpression and VIGS

**Figure 5:**
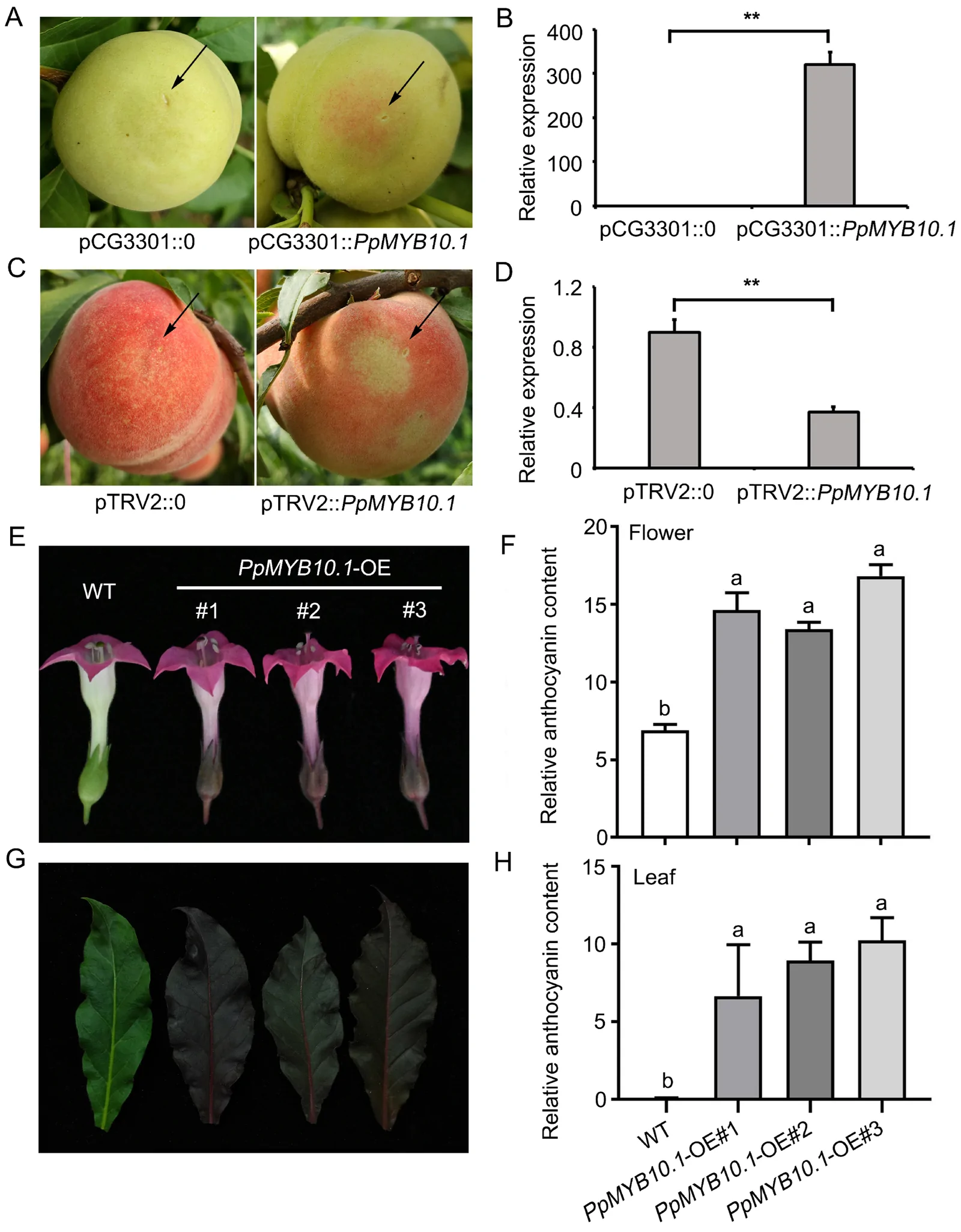
Functional validation of *PpMYB10.1*. (**A**) Transient overexpression and (**B**) RT-qPCR in non-red peach fruit. (**C**) VIGS and (**D**) RT-qPCR in red peach fruit. The black arrow indicates the injection site. (**E**) Overexpression and (**F**) total anthocyanin content in tobacco flowers. (**G**) Overexpression and (**H**) total anthocyanin content in tobacco leaves. Total anthocyanin content was measured by the spectrophotometric method and is expressed as absorbance units (A_530_ − 0.25A_657_) per gram fresh weight (g^−1^ FW), representing relative anthocyanin quantification. Error bars represent standard deviation (*n* = 3 biological replicates). Different letters above bars indicate significant differences (*p* < 0.05, one-way ANOVA followed by Tukey’s post hoc test), while asterisks indicate significant differences between treatment and control groups (** *p* < 0.01, Student’s *t*-test).

To validate the gene function of *PpMYB10.1*, the full-length coding sequence from the non-red skin type was ligated into a pCG3301 plasmid under the control of the CaMV 35S promoter and injected into non-red fruit for transient overexpression, as well as into tobacco leaves and flowers for stable overexpression. After injection for 8–10 days, the fruit skin of the non-red type showed phenotypic complementation, resulting in anthocyanin accumulation and a red skin phenotype (Figure 5A). RT-qPCR showed a dramatic increase in *PpMYB10.1* expression in the transgenic fruit skin relative to the control (CK) (Figure 5B). To further validate the gene function, we silenced *PpMYB10.1* in the 5′ region of red skin fruit using VIGS. At the injection sites where the gene was silenced, anthocyanin accumulation was blocked, and the fruit showed a faded red phenotype (Figure 5C). RT-qPCR confirmed that *PpMYB10.1* expression was lower than in the CK (Figure 5D). We also generated 35S:*PpMYB10.1* transgenic tobacco plants, which showed significantly enhanced anthocyanin accumulation throughout the plant, including leaves and flowers (Figure 5E,G), with significantly increased gene expression compared to wild-type plants (Figure 5F,H). Together, these results demonstrate that *PpMYB10.1* functions as a positive regulator of anthocyanin biosynthesis.

### 2.6. Promoter Activity Assay and Subcellular Localization of PpMYB10.1

**Figure 6:**
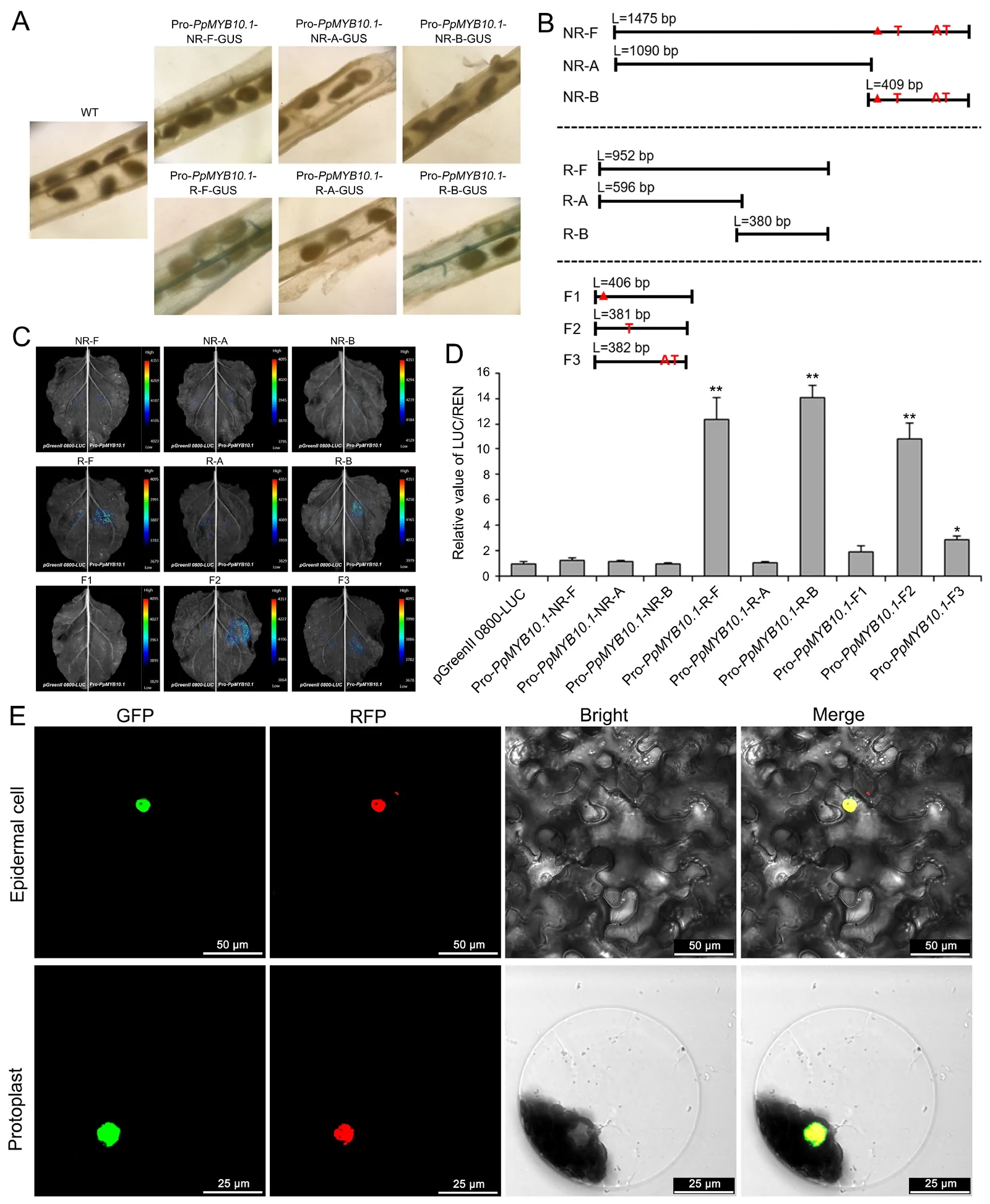
Promoter activity and subcellular localization of *PpMYB10.1*. (**A**) GUS activity in *Arabidopsis* siliques. (**B**) L indicates the length of each promoter fragment. (**C**) LUC imaging and (**D**) relative LUC/REN ratio of promoter constructs. (**E**) Subcellular localization in epidermal cells (Bar = 50 μm) and protoplasts (Bar = 25 μm). Values are means ± SD (*n* = 3). Asterisks indicate significant differences in LUC/REN ratio between indicated pairs (Student’s *t*-test, * *p* < 0.05, ** *p* < 0.01). Red triangle, red A, and red AT denote the three insertion variants between red- and non-red-skin promoters (26-bp, 1-bp, and 2-bp insertions, respectively). R-F, red-type full-length promoter (952 bp); NR-F, non-red full-length promoter (1475 bp); R-A/NR-A and R-B/NR-B, 5′ and 3′ sub-fragments; F1, synthesized fragment containing only the 26-bp insertion; F2, synthesized fragment without the 26-bp insertion but containing the 1-bp insertion; F3, synthesized fragment without the 26-bp insertion but containing the 2-bp insertion.

Transgenic *Arabidopsis* lines were used to determine the variation in the *PpMYB10.1* promoter region that determines transcriptional and spatial activity. Primers were designed to amplify the promoter region, yielding 952 bp for red and 1475 bp for non-red skin-type fruit (Figures S2 and S3, Table S5). GUS histochemical assay of T1 *Arabidopsis* lines showed that the red-type promoter drove GUS expression in siliques, whereas the non-red-type promoter showed no detectable GUS activity (Figure 6A). To pinpoint the core functional regions, a full-length promoter (F), two sub-fragments (A and B), and three synthesized fragments (F1, F2, F3) corresponding to the 26-bp, 1-bp, and 2-bp insertion alleles, respectively, were tested (Figure 6B). To exclude potential functional contributions from the 494-bp InDel and six SNPs located in the 5′ region, we compared the activity of the 5′ sub-fragments (R-A vs. NR-A), which contain the 494-bp InDel and six SNPs but lack the 26-bp, 1-bp, and 2-bp insertions. Both R-A and NR-A showed negligible promoter activity, indicating that the 5′ region is not the critical promoter region and that the 494-bp InDel and six SNPs are not functional variants (Figure 6C,D). In contrast, the 3′ sub-fragments (R-B vs. NR-B), which contain the 26-bp, 1-bp, and 2-bp insertions but lack the 494-bp InDel and six SNPs, showed clear activity differences (R-B strong vs. NR-B weak), localizing the functional variants to the 3′ region (Figure 6C,D). Together with the synthesized fragment experiments showing that removal of the 26-bp insertion is sufficient to restore promoter activity regardless of the 1-bp or 2-bp insertions, these results demonstrate that the 26-bp insertion is the core functional polymorphism (Figure 6C,D). Furthermore, the PpMYB10.1::GFP fusion protein co-localized with the nuclear marker in both tobacco leaf epidermal cells and protoplasts (Figure 6E), consistent with its function as a transcription factor.

### 2.7. Development of a Co-Segregation InDel Marker

By analyzing the RNA-seq data of *PpMYB10.1*, three exons were annotated, and one 6-bp deletion and two SNPs were identified in the coding region (Figure S1). Nucleotide sequence analysis of the upstream regions identified ten polymorphic sites, comprising four InDels (494-bp, 26-bp, 2-bp, and 1-bp) and six SNPs (Figure S2). To evaluate potential associations between these variations and anthocyanin deficiency, we performed whole-genome resequencing of 22 peach cultivars. Genotyping revealed that only the 26-bp, 2-bp, and 1-bp insertions exhibited complete linkage disequilibrium with fruit skin color. Based on the promoter activity assays, we hypothesized that the 26-bp insertion disrupts transcriptional regulation, leading to suppressed *PpMYB10.1* expression in non-red skin. We therefore developed a co-dominant molecular marker targeting the 26-bp InDel locus and genotyped 126 peach accessions and three segregating populations (Table S6). The 26-bp insertion/deletion polymorphism exhibited complete linkage with the non-red/red fruit skin phenotype (Tables S7 and S8), indicating that this InDel is an effective molecular marker for distinguishing skin color in peach breeding. The number of individuals and the genotype-phenotype concordance for each validation panel are summarized in Table 1. These three populations were derived from ‘Zhongtaobaiyu’ crossed with three different other parents (‘7-4-21’, ‘7-7-75’, and ‘04-7-13’); although ‘Zhongtaobaiyu’ was also used in the mapping cross with ‘09-bei8-25’, the validation progeny are distinct individuals from different cross combinations and were not among the 180 BC1 individuals used for fine mapping.

## 3. Discussion

Bulked segregant analysis coupled with whole-genome resequencing (BSA-seq) is a cost-effective strategy for rapidly anchoring qualitative traits in segregating populations [7,29,30,31,32]. In this study, using a BC1 population segregating for fruit skin color, the expected Δ(SNP-index) between the red- and non-red-pools was 0.5, which allowed the color locus to be anchored to chromosome 3 in a single mapping round. Subsequent fine mapping with six recombinant individuals delimited the interval to 453.083 kb, and integration of RNA-seq data pinpointed *PpMYB10.1* as the only gene within this region that was strongly and consistently differentially expressed between the two color groups. The present study instead points to a 26-bp insertion in the *PpMYB10.1* promoter as an associated variant: reporter assays showed that this insertion strongly reduces promoter activity, and *PpMYB10.1* expression was below the detection limit in non-red skin, suggesting that promoter structural variation may contribute to the non-red skin phenotype. The MYB gene family is extensive and functionally diverse, with members such as *PpMYB10.1*, *PpMYB10.2*, *PpMYB10.3*, and *PpMYB10.4* playing roles in anthocyanin accumulation in fruits [14,15,19]. MYB genes regulate leaf, flower, fruit flesh, and skin anthocyanin levels across diverse plants, including apple flesh [23,33,34,35], grape skin [36], pear skin [37], strawberry flesh [38], sweet cherry flesh [39], tea (*Camellia sinensis*) leaf [40], and peach leaf, flower, and flesh [5,41,42]. For peach, Rahim et al. [19] reported that the expression levels of *PpMYB10.1* and *PpMYB10.3* correlated with anthocyanin content in the peel and mesocarp.

Our RNA-seq and targeted metabolomic data allow a more integrated view. Experimentally, non-red skin showed near-complete loss of *PpMYB10.1* transcripts together with coordinated down-regulation of structural genes in the anthocyanin pathway—*PAL*, *CHS*, *F3H*, *DFR*, *ANS*, *UFGT*, and the anthocyanin transporter *PpGST*—and a near-absence of detectable anthocyanins. Based on the conserved MYB–bHLH–WD40 regulatory module in plants, we propose that the reduced *PpMYB10.1* transcript level drives this down-regulation; this link is inferred rather than directly proven by genetic complementation in peach. We further observed that certain flavonoid branches, notably procyanidin A1/A2 and some malvidin derivatives, were elevated in non-red skin. One possible explanation is that, once anthocyanin synthesis is blocked, phenylpropanoid flux is redirected toward competing proanthocyanidin and flavonol branches; however, this redirection remains a hypothesis that would require flux analysis or isotope tracing to confirm. Other mechanisms may also contribute, including altered vacuolar transport of anthocyanins by the down-regulated *PpGST*, post-transcriptional regulation by microRNAs or by redundant *PpMYB10.2–10.4* paralogs, and upstream regulators of *PpMYB10.1* that were not directly tested here. We therefore distinguish throughout between relationships supported by our assays (promoter activity, expression, metabolite levels) and proposed mechanistic links that warrant future validation.

It is important to note that our evidence consists of two independent lines of inquiry. First, the transient overexpression and VIGS experiments validate the gene function of *PpMYB10.1* as a positive regulator of anthocyanin biosynthesis. Second, the promoter activity assays, particularly the synthesized fragment experiments comparing F1 (with the 26-bp insertion) and F2/F3 (without the 26-bp insertion), provide the most direct evidence for the functional role of the 26-bp insertion in suppressing promoter activity. Together, these two lines of evidence establish that *PpMYB10.1* is a positive regulator of anthocyanin biosynthesis, and that the 26-bp promoter insertion reduces its expression, leading to the non-red skin phenotype. While the present study provides strong evidence supporting the association between the 26-bp promoter insertion and anthocyanin deficiency, several avenues for further validation remain. First, the synthesized fragment experiments differing exclusively by the 26-bp insertion strongly support its functional effect; targeted genome editing in peach would further strengthen the causal link in future work. Second, promoter activity was evaluated in heterologous systems (*Arabidopsis* and *N. benthamiana*), which serve as efficient preliminary platforms; direct validation in peach fruit epidermal tissues would provide additional physiological relevance. Third, anthocyanin content was measured by spectrophotometric method as relative absorbance units; absolute quantification using authentic standards would enable more precise comparisons. These complementary approaches will further refine our understanding of the regulatory mechanism.

Fruit skin color is a key commercial trait in peach. In China and Asia, growers commonly bag fruit to inhibit anthocyanin accumulation and produce yellow or white phenotypes, which increases production costs [43,44]. Therefore, breeding cultivars with naturally anthocyanin-deficient skin through marker-assisted selection (MAS) can accelerate such programs. In the present study, we developed a co-dominant InDel marker that targets the 26-bp insertion in the *PpMYB10.1* promoter and validated it in 126 peach accessions and three segregating hybrid populations, where it co-segregated with skin color. This marker should enable early prediction of skin color in seedlings, complementing previously reported markers for red-fleshed peach. The 26-bp insertion itself also represents a candidate target for precision genome editing, although editing in peach remains technically demanding. Collectively, our BSA-seq, RNA-seq, and resequencing data point to *PpMYB10.1* as a key regulator of anthocyanin biosynthesis in peach fruit skin, with a 26-bp promoter insertion associated with reduced promoter activity, suppressed gene expression, and anthocyanin deficiency. Future work will address the broader regulatory network of *PpMYB10.1* and confirm causality through targeted editing in peach.

## 4. Materials and Methods

### 4.1. Plant Materials

Three peach accessions with different skin color types were used for hybridization. They included ‘03-33-7’, ‘09-bei8-25’, and ‘Zhongtaobaiyu’. ‘03-33-7’ is a premium nectarine variety characterized by its vibrant, entirely red fruit skin (Figure 1A). ‘09-bei8-25’ is an F1 hybrid of the cross between ‘03-33-7’ and ‘Zhongtaobaiyu’, which exhibits partially red fruit skin (Figure 1B). ‘Zhongtaobaiyu’ is a variety that displays anthocyanin deficiency only in its fruit skin (Figure 1C). The BC1 segregation population of 180 individuals was derived from the cross ‘09-bei8-25’ × ‘Zhongtaobaiyu’. All materials were maintained at the experimental station of the Zhengzhou Fruit Research Institute in Xinxiang, Henan, China (35°11′ N, 113°47′ E). For BSA-seq analysis, 20 red skin type and 20 non-red skin type BC1 individuals were used to generate two mixed gene pools. For fine mapping, the 180 BC1 individuals were used to screen for recombinants and narrow the genetic interval. For transcriptomic and metabolomic analyses, three biological replicates were used. Each replicate consisted of peel tissue pooled from 3–5 fruits harvested from three different trees of the same genotype, grafted on the same rootstock and grown in the same orchard block, so that the three replicates are biologically independent rather than technical repeats. All fruits were harvested at the breaker stage, based on firmness and skin background color. Only fruits exposed to direct sunlight on the outer canopy at a similar height were sampled; bagged or shaded fruits were excluded. Red- and non-red-skin samples were picked on the same day, from adjacent rows, under the same weather conditions, and were immediately peeled on ice and frozen in liquid nitrogen.

### 4.2. Anthocyanin Content Analysis Using HPLC-MS/MS

Freeze-dried fruit skin was powdered using a mixer mill (MM 400; Retsch, Haan, Germany) with zirconia beads for 1.5 min at 30 Hz. Next, 100 mg of the powder was extracted at 4 °C overnight using 1.0 mL of 70% aqueous methanol. The extracts were centrifuged at 10,000× *g* for 10 min, absorbed using a CNWBOND Carbon-GCB SPE Cartridge (250 mg, 3 mL; ANPEL, Shanghai, China), and filtered through a SCAA-104 membrane (0.22-μm pore size; ANPEL, Shanghai, China) before LC-MS analysis.

The extracts were analyzed using an HPLC-ESI-MS/MS system (HPLC, Shim-pack UFLC SHIMADZU CBM30A system, Shimadzu, Kyoto, Japan; MS, Applied Biosystems 6500 Q TRAP, Applied Biosystems, Foster City, CA, USA). The analytical conditions were as follows: column, Waters ACQUITY UPLC HSS T3 C18 (1.8 μm, 2.1 mm × 100 mm; Waters Corporation, Milford, MA, USA); solvent system, water (0.04% acetic acid): acetonitrile (0.04% acetic acid); gradient program, 100:0 (*v*/*v*) at 0 min, 5:95 (*v*/*v*) at 11.0 min, 5:95 (*v*/*v*) at 12.0 min, 95:5 (*v*/*v*) at 12.1 min, 95:5 (*v*/*v*) at 15.0 min; flow rate, 0.40 mL/min; temperature, 40 °C; injection volume, 2 μL. The effluent was alternately connected to an ESI-triple quadrupole-linear ion trap (Q TRAP)-MS (SCIEX, Framingham, MA, USA).

For ESI-Q TRAP-MS/MS, the ESI source operation parameters were set as follows: turbo spray ion source; source temperature 500 °C; ion spray voltage 5500 V; and ion source gas I (GSI), gas II (GSII), and curtain gas (CUR) set at 55, 60, and 25.0 psi, respectively. Instrument tuning and mass calibration were performed using 10 and 100 μmol/L polypropylene glycol solutions in QQQ and LIT modes, respectively. QQQ scans were acquired as MRM experiments with the collision gas (nitrogen) set to 5 psi. DP and CE for the MRM transitions were optimized individually. A specific set of MRM transitions was monitored for each period according to the metabolites eluted within that period. The data were processed using the criteria of |log2(fold change)| ≥ 1 and *p* < 0.05.

Anthocyanin and related flavonoid compounds were quantified using multiple reaction monitoring (MRM) mode in positive ion mode. Detailed MRM parameters for all 11 detected compounds are provided in Table S9. The reported abundances represent relative peak areas normalized to internal standards. Fold changes were calculated as the ratio of mean abundances between red and non-red groups. Absolute quantification was not performed due to the lack of commercially available standards for all compounds (Table S10).

### 4.3. Genomic DNA Extraction and BSA-Seq Analysis

For resequencing, genomic DNA (gDNA) of the parents (‘09-bei8-25’ and ‘Zhongtaobaiyu’) and 20 individuals of each skin type (red and non-red) for BSA-seq were extracted using the cetyltrimethylammonium bromide (CTAB) method [45]. For fine mapping, a high-throughput gDNA extraction method was performed for all offspring using the CTAB method with modifications for SNP and InDel polymorphism genotyping [46]. gDNA was quantified using a NanoDrop spectrophotometer (Thermo Fisher Scientific, Waltham, MA, USA). In total, 20 DNA samples of the red and non-red skin types were mixed in equal ratios to generate DNA pools for each skin type. These pools were then sequenced on an Illumina HiSeq™ PE150 (Illumina, San Diego, CA, USA) by Novogene Co., Ltd. (Beijing, China).

After generating clean data, the reads were aligned to the peach reference genome using the BWA aligner [47]. SAMtools was used to convert the alignment files, conduct SNP analysis, remove duplicates, and annotate the detected SNPs [48]. GATK 3.3 UnifiedGenotyper and VariantFiltration were used to filter SNPs using the following settings: QD < 4.0, FS > 60.0, MQ < 40, and GQ < 20 [49]. To further remove low-quality variants, SNPs with SNP-index < 0.3 in both pools, depth < 7, or missing data in one pool were excluded from analysis. The SNP-index was calculated for the non-red and red skin pools following the method of Abe et al. [29] and Lu et al. [7]. Δ(SNP-index) was calculated as the difference in SNP-index between the non-red and red skin pools. The 95% confidence interval for Δ(SNP-index) was estimated by simulation under the null hypothesis of no linkage. To eliminate sequencing errors, the two parents, ‘Zhongtaobaiyu’ and ‘09-bei8-25’, were included in the analysis. Each DNA pool was analyzed using a 1-Mb sliding window with a 1-kb step size to determine the candidate regions associated with the fruit skin color trait. Candidate regions identified by BSA-seq were further refined using PCR-based SNP and InDel markers; six recombinant individuals were identified among the 180 BC1 progeny, and their recombination breakpoints defined the final 453.083-kb mapping interval. All genomic coordinates refer to the peach reference genome v2.0.

### 4.4. SNP and InDel Marker Development for Fine Mapping and Co-Segregation Analysis

In the mapping region, SNP and InDel markers were designed based on the resequencing data of the parents ‘Zhongtaobaiyu’ and ‘09-bei8-25’. The BAM files were loaded into Integrative Genomics Viewer (IGV) software (version 2.19.8) [50] to develop homozygous SNPs or InDels in ‘Zhongtaobaiyu’ and heterozygous ones in ‘09-bei8-25’ corresponding to the phenotypes. Primers were designed to span the SNP or InDel sites based on the resequencing data of the parents using Primer3. PCR products in a 40-μL reaction volume were genotyped using a Sanger sequencer (ABI; Applied Biosystems, Foster City, CA, USA) to detect SNPs and electrophoresed on a PAGE gel in a 15-μL reaction volume to detect InDels. Genotyping data were collected and analyzed in Microsoft Excel (Microsoft Corporation, Redmond, WA, USA) to narrow down the mapping region.

### 4.5. RNA Extraction and GENE Expression Analysis Using RNA-Seq

Total RNA was isolated using a TRIzol Total RNA Extractor kit (Sangon, Shanghai, China) following the manufacturer’s instructions. The total RNA was purified and assessed for quality. mRNA was enriched with oligo(dT) and then sheared into small fragments using random hexamer-primed reverse transcription. First-strand cDNA was then used to generate second-strand cDNA. After adapter addition, PCR amplification, purification, and quality control, the double-stranded PCR products were denatured and circularized using a splint oligo sequence to obtain the final library. Single-end 50-base reads were generated using a BGIseq500 platform (BGI-Shenzhen, Shenzhen, China).

Raw data were filtered to obtain clean data, and the reads were aligned to the peach reference genome v2.0, followed by calculation of fragments per kilobase of transcript per million mapped reads (FPKM) (Table S11). Differentially expressed genes were identified between non-red skin and red skin types using the DESeq2 package in R (version 4.5.3) (R Foundation for Statistical Computing, Vienna, Austria). DESeq2 normalization used the default median-of-ratios method, and differential expression was tested by a Wald test. An adjusted *p*-value of 0.05 and a |log2(fold change)| ≥ 1 were set as the thresholds for significant differential expression.

### 4.6. Plasmid Construction for VIRUS-Induced Gene Silencing (VIGS)

For VIGS analysis, a 281-bp fragment from the 5′ region of *PpMYB10.1* was amplified using the cDNA of red skin type fruit as the template and ligated into TRV2 using a ClonExpress II One Step Cloning Kit (Vazyme, Nanjing, China), according to the manufacturer’s instructions. TRV1, TRV2, and TRV2-*PpMYB10.1* were transferred into Agrobacterium tumefaciens GV3101 and incubated at 28 °C in LB medium to an OD600 of 0.8–1.0. The precipitate was resuspended in Agrobacterium infiltration buffer (10 mmol L^−1^ MgCl_2_, 10 mmol L^−1^ MES, and 200 μmol L^−1^ acetosyringone, pH 5.6) after centrifugation at 8000 rpm to reach an OD600 between 0.8 and 1.0. Subsequently, pTRV2 or pTRV2-*PpMYB10.1*, together with pTRV1, were mixed in a 1:1 ratio. After incubation for 3 h, the mixture was injected into red skin fruits at the breaker stage. More than 30 fruits were injected in three biological replicates. After Agrobacterium infiltration, injected fruits were incubated in a growth chamber at 25 °C with a 16-h light/8-h dark photoperiod and a light intensity of approximately 200 μmol m^−2^ s^−1^ for 8–10 days prior to phenotyping and sampling.

### 4.7. Plasmid Construction for Transient Gene Overexpression Analysis

For transient overexpression, the full-length coding sequence of *PpMYB10.1* was amplified using the cDNA template of red skin type fruit (‘09-bei8-25’) and then ligated into a pCG3301 vector using a ClonExpress II One Step Cloning Kit (Vazyme, Nanjing, China) according to the manufacturer’s instructions. After confirmation of the ligated sequence, the vector was transferred into A. tumefaciens GV3101 and incubated at 28 °C until an OD600 of 0.8–1.0 was reached. After centrifugation at 8000 rpm, the precipitate was resuspended in Agrobacterium infiltration buffer (10 mmol L^−1^ MgCl_2_, 10 mmol L^−1^ MES, and 200 μmol L^−1^ acetosyringone, pH 5.6) to an OD600 of 0.8–1.0. More than 30 fruits were injected in three biological replicates. The full coding sequence of *PpMYB10.1* was amplified and cloned into a pBI121 vector. The recombinant plasmids were then transformed into A. tumefaciens GV3101 for *Nicotiana tabacum* transgenic manipulation using the leaf disk method [51]. Positive transgenic plants were selected on 50 mg/L kanamycin and self-pollinated twice to generate T2 transgenic progeny. *PpMYB10.1*-OE transgenic lines showing obvious color changes in leaves and flowers were used for further analysis. Following injection, fruits were maintained under the same conditions as VIGS (25 °C, 16-h light/8-h dark, 200 μmol m^−2^ s^−1^) for 8–10 days before anthocyanin phenotyping and sample collection.

### 4.8. Real-Time Quantitative Reverse Transcription-PCR Analysis

Total RNA of fruit peel was extracted using a TRIzol Total RNA Extractor kit (Sangon, Shanghai, China) following the manufacturer’s instructions for RT-qPCR analysis. First-strand cDNA was synthesized using a RevertAid First-Strand cDNA Synthesis Kit (Thermo Fisher Scientific, Waltham, MA, USA) according to the manufacturer’s instructions. A reaction volume of 15 μL containing 1× SYBR Green I Master Mix (Roche, Basel, Switzerland), 30 ng cDNA, and 200 nM of each primer was run on a LightCycler 480 II instrument (Roche). The program was initiated with 3 min at 95 °C, followed by 45 cycles of 95 °C for 10 s, 60 °C for 20 s, and 72 °C for 10 s. For each cycle, the fluorescence intensity was detected at 60 °C. Melting curves were generated at the end of 45 cycles from 60 °C to 90 °C at a ramp rate of 0.03 °C/s. The translation elongation factor gene *PpTEF2* was selected as the reference gene (GenBank accession no. JQ732180) [52]. The primers 5′-CTCAGTTCCTCTCCCGTCAGCC-3′ and 5′-GCAGCACCAGAAGTTGAAGTACCA-3′ for *PpMYB10.1* were designed with a fragment size of 237 bp for RT-qPCR. The suitability of *PpTEF2* as a reference gene was verified by its stable expression across all samples and treatments (Cq variation < 1 cycle). Relative expression was calculated using the 2^−ΔΔCt^ method, with amplification efficiencies between 90% and 110% for both target and reference genes. Amplicon specificity was confirmed by a single peak in melting curve analysis and a single band on agarose gel. Three biological and three technical replicates were performed for each sample.

### 4.9. GUS Expression and Staining of Transgenic Arabidopsis for Promoter Identification

The candidate promoter regions of *PpMYB10.1*—1475 bp upstream of the start codon (ATG) in non-red skin type ‘Zhongtaobaiyu’ and 952 bp in red skin type fruit—were amplified and ligated into pBI121 harboring β-glucuronidase (GUS) as a reporter gene (pBI121::GUS) using a ClonExpress II One Step Cloning Kit (Vazyme, Nanjing China) according to the manufacturer’s instructions. The constructs were transformed into *Arabidopsis* (Columbia, Col-0) as described previously [53]. A histochemical assay for GUS activity was conducted. Briefly, *Arabidopsis thaliana* tissues were sectioned using a scalpel, and the roots, leaves, and siliques were incubated in staining buffer from a GUS Stain Kit (Coolaber, Beijing, China) at 37 °C in the dark for 24 h. The histochemical staining of GUS activity in T1 *Arabidopsis* was performed according to the manufacturer’s instructions. Thereafter, the tissues were rinsed several times with 70% (*v*/*v*) ethanol and examined under a stereo microscope (SZX10, Olympus, Tokyo, Japan).

### 4.10. Promoter Activity Testing Using the Dual-Luciferase Assay

A dual-luciferase (LUC) assay was employed to evaluate the promoter activity [54]. The full-length promoter of *PpMYB10.1* and two different segments (A and B) for non-red (NR) and red (R) fruit skin types, including pro-*PpMYB10.1*-R/NR-F, pro-*PpMYB10.1*-R/NR-A, and pro-*PpMYB10.1*-R/NR-B, were amplified and inserted into a pGreenII 0800-LUC vector. In addition, three synthesized fragments (F1, F2, and F3), corresponding to the 26-bp insertion allele, the 2-bp insertion allele, and the 1-bp insertion allele, respectively, were generated. All constructs were transformed into *A. tumefaciens* strain GV3101. A dual-luciferase assay was performed using *Nicotiana benthamiana* leaves. Agrobacterium cultures carrying the constructs were resuspended in infiltration buffer (10 mM MES, 10 mM MgCl_2_, and 150 mM acetosyringone, pH 5.6) to an OD600 of approximately 0.75. The Agrobacterium suspensions were infiltrated individually into tobacco leaves. Firefly and Renilla luciferase activities were assayed 3 days after infiltration using dual-luciferase assay reagents (Promega, Madison, WI, USA). The assay was performed in three independent experiments with three biological replicates.

### 4.11. Subcellular Localization of PpMYB10.1

The full-length coding sequence of *PpMYB10.1* without the stop codon was amplified by PCR using cDNA from red or non-red skin-type fruit. The resulting product was ligated into pBI221 carrying the green fluorescent protein (GFP) gene driven by CaMV 35S, using a ClonExpress II One Step Cloning Kit (Vazyme, Nanjing, China) according to the manufacturer’s instructions. An empty pBI221 vector was used as the control (CK). After sequence confirmation and purification, 1 μg/μL plasmid DNA was used for transfection. Three-week-old tobacco (*N. benthamiana*) leaves were infiltrated using syringes and used for protoplast isolation. Tobacco protoplast isolation and transfection were performed as described by Yoo et al. [55]. An empty GFP vector and mCherry were used as nuclear markers. Both red (RFP) and green (GFP) fluorescent protein signals in the epidermal cells and protoplasts of tobacco leaves were observed using a confocal microscope (Leica TCS SP3; Leica Microsystems, Wetzlar, Germany).

### 4.12. Statistical Analyses

All data were analyzed using either Student’s *t*-test for two-group comparisons or one-way ANOVA followed by Tukey’s post hoc test for multiple group comparisons. False discovery rate (FDR) correction was applied for multiple testing where applicable. Differences were considered significant at *p* < 0.05. All statistical analyses were performed using R (version 4.5.3) (R Foundation for Statistical Computing, Vienna, Austria). Graphs were generated using Origin 9.1 (MicroCal Software, Inc., Northampton, MA, USA).

## 5. Conclusions

In this study, we fine-mapped the peach fruit skin color locus to a 453.083-kb interval on chromosome 3 and, integrating BSA-seq, resequencing, and RNA-seq data, identified *PpMYB10.1* as a strong candidate. Transient overexpression and VIGS confirmed the positive regulatory role of *PpMYB10.1* in anthocyanin biosynthesis, while GUS and dual-luciferase reporter assays indicated that a 26-bp insertion in the *PpMYB10.1* promoter is associated with strongly reduced promoter activity and near-absent gene expression in non-red skin. A co-dominant InDel marker targeting this insertion was developed and validated in 126 accessions and three hybrid populations, providing a practical tool for marker-assisted seedling selection. We note that causality of the 26-bp insertion would benefit from targeted genome editing in peach, which remains technically challenging.

## Figures and Tables

**Table 1:** Summary of marker validation results across all tested materials.

Accessions/Populations	Total No.	Consistent/Total	Concordance
Peach accessions	126	126/126	100%
‘Zhongtaobaiyu’ × ‘7-4-21’	32	32/32	100%
‘Zhongtaobaiyu’ × ‘7-7-75’	68	68/68	100%
‘Zhongtaobaiyu’ × ‘04-7-13’	168	168/168	100%

## Data Availability

The whole-genome re-sequencing data of 22 peach cultivars and the BSA-seq data have been deposited in the NCBI SRA database under accession number PRJNA1177659, and the RNA-seq data have been deposited under accession number PRJNA1177642. Processed data are provided in the Supplementary Materials: genotyping and co-segregation datasets are summarized in Tables S2 and S7; metabolite identification parameters and peak areas of the 11 anthocyanin/proanthocyanidin compounds are provided in Tables S9 and S10; the full gene-level expression matrix (FPKM, log2 fold change, raw *p*-value, and FDR-adjusted *p*-value) is provided in Table S11. All key analytical parameters are specified in Section 4. Analysis scripts and computational parameters are available from the corresponding author upon reasonable request.

## Supplementary Materials

The following supporting information can be downloaded at: https://www.mdpi.com/article/10.3390/ijms272110027/s1.

## Abbreviations

The following abbreviations are used in this manuscript:

BSA-seq, bulked segregant analysis sequencing; C4H, cinnamate 4-hydroxylase; CHS, chalcone synthase; CTAB, cetyltrimethylammonium bromide; DFR, dihydroflavonol 4-reductase; FLS, flavonol synthase; FPKM, fragments per kilobase of transcript per million mapped reads; F3H, flavanone 3-hydroxylase; GFP, green fluorescent protein; gDNA, genomic DNA; GST, glutathione S-transferase; GUS, β-glucuronidase; HPLC-MS/MS, high-performance liquid chromatography-tandem mass spectrometry; IGV, Integrative Genomics Viewer; InDel, insertion/deletion; LUC, luciferase; MBW, MYB-bHLH-WD40; PAL, phenylalanine ammonia-lyase; ANS, anthocyanidin synthase; RT-qPCR, real-time quantitative reverse transcription PCR; RNA-seq, RNA sequencing; SNP, single nucleotide polymorphism; SSR, simple sequence repeat; UFGT, UDP-glucose:flavonoid 3-O-glucosyltransferase; UGT, UDP-glycosyltransferase; VIGS, virus-induced gene silencing.
