# Supplementary material for: A Novel 26-bp Insertion in the PpMYB10.1 Promoter Is Associated with Anthocyanin Deficiency in Peach (Prunus persica) Fruit Skin
Source: Int J Mol Sci. 2026 Nov 9;27(21):10027. doi: 10.3390/ijms272110027 (PMC13764401; doi:10.3390/ijms272110027)
Supplement: Supplementary file 1 [file ijms-27-10027-s001.zip › Supplemental figures.pdf]

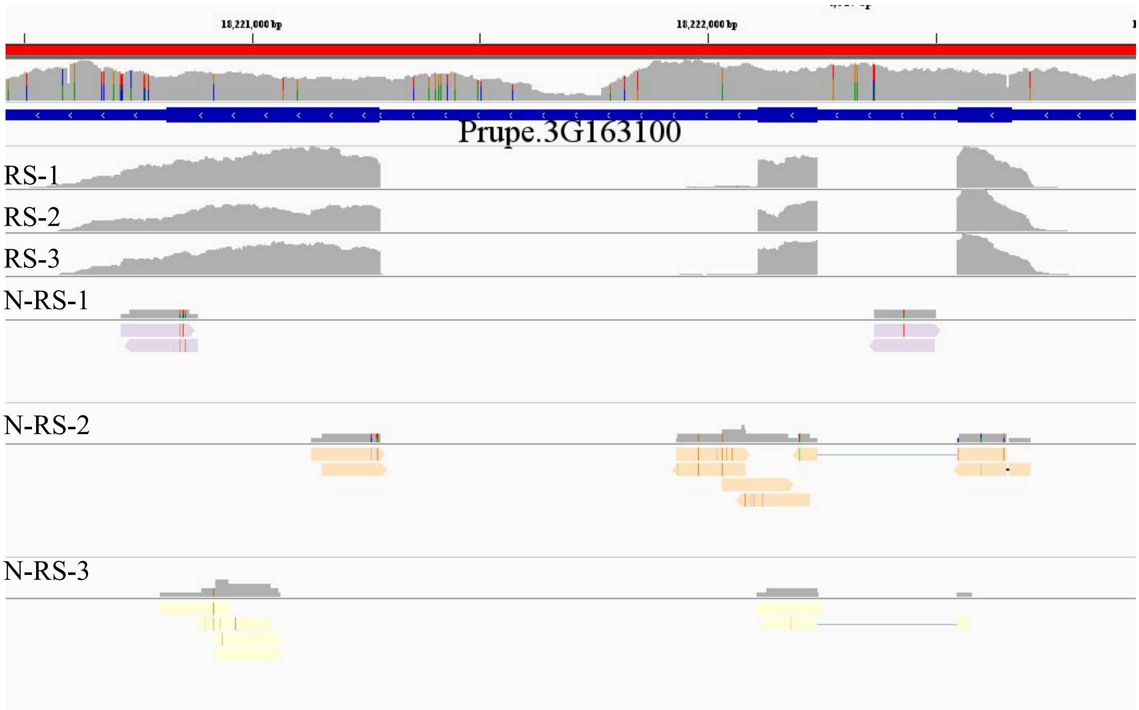


**Figure S1. RNA-seq data mapping result of *PpMYB10.1* viewed in the IGV software.** RS-1, RS-2 and RS-3 are three biological replicates data (FPKM) of the red fruit skin type; N-RS-1, N-RS-2 and N-RS-3 are three biological replicates data (FPKM) of the non-red fruit skin type.

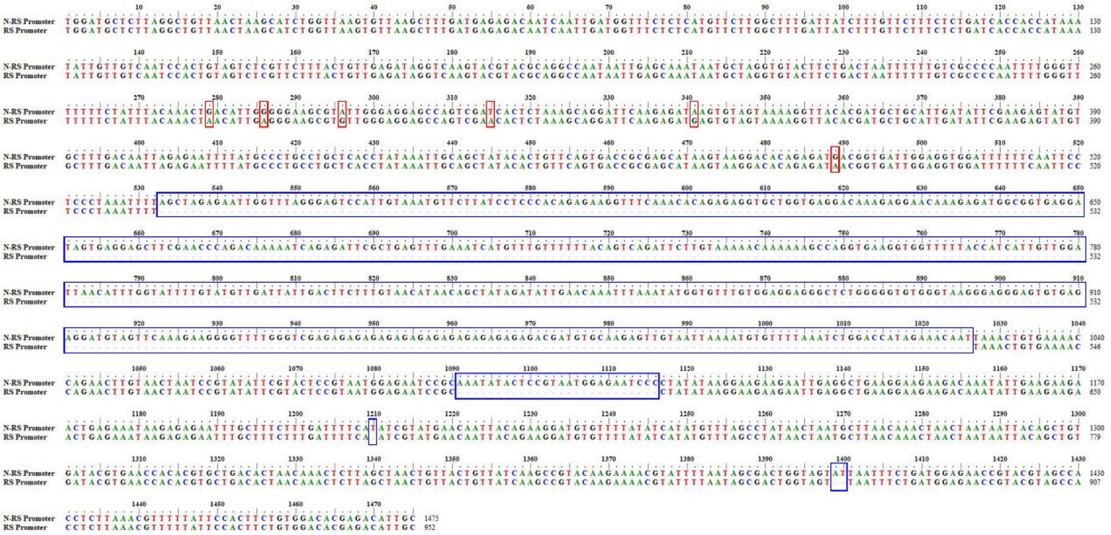

**Figure S2. Sequence alignment of the promoter regions in the red and non-red fruit skin type.** N-RS, non-red skin. RS, red skin. Red rectangles indicate the six SNPs. Blue rectangles indicate the four InDels.

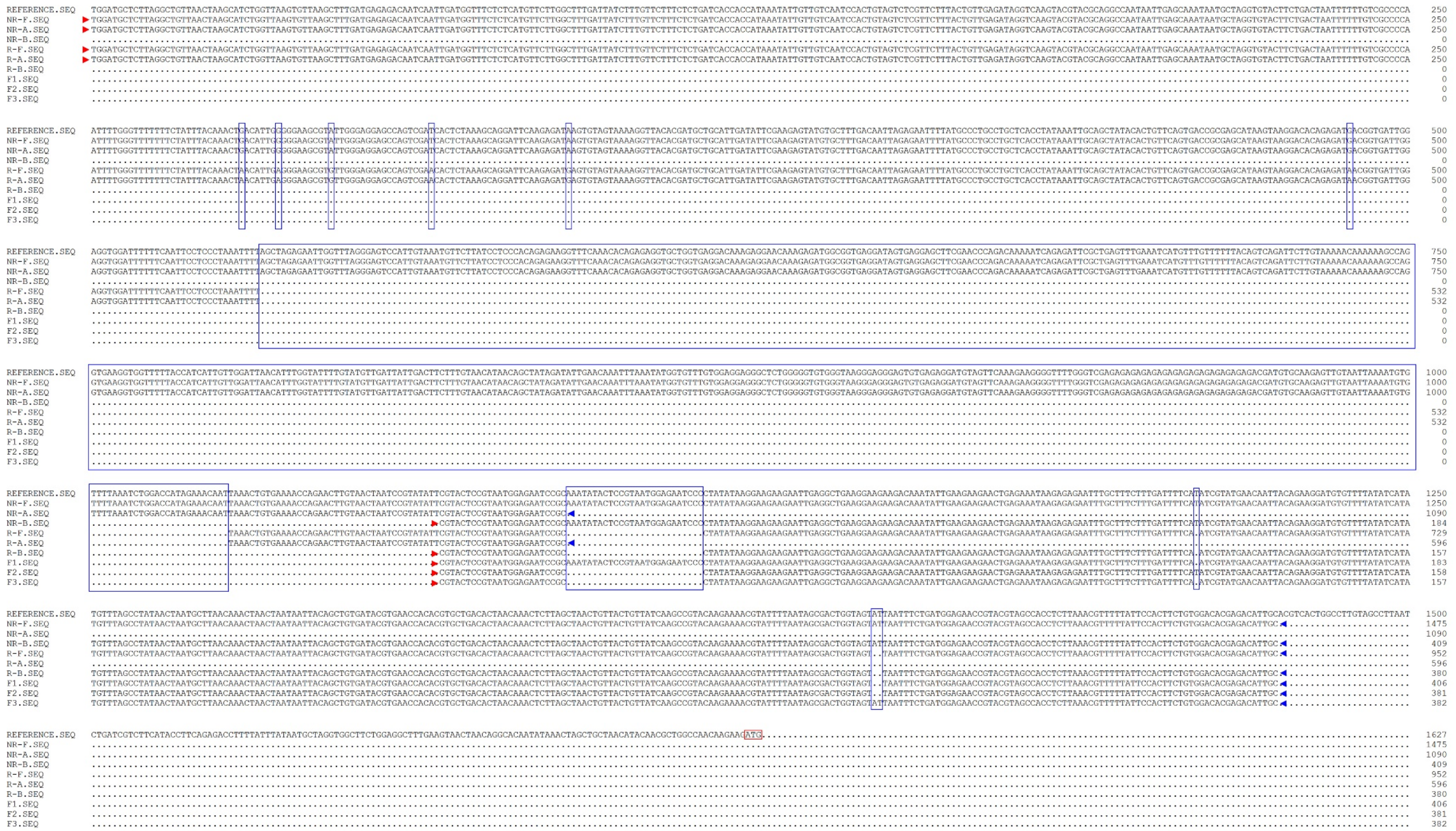
REFERENCE.SEQ
NR-F.SEQ
NR-A.SEQ
NR-B.SEQ
R-F.SEQ
R-A.SEQ
R-B.SEQ
F1.SEQ
F2.SEQ
F3.SEQ
1627

**Figure S3. Multiple sequence alignment of the *PpMYB10.1* promoter fragments used in this study.** The peach reference promoter sequence is shown at the top (labeled REFERENCE). N-RS-F denotes the non-red-skin full-length promoter; N-RS-A and N-RS-B denote the 5′and 3′sub-fragments of the non-red-skin promoter; R-F denotes the red-skin full-length promoter; R-A and R-B denote the 5′and 3′sub-fragments of the red-skin promoter; F1, F2 and F3 denote the synthesized promoter fragments. Red arrowheads indicate the left boundaries and blue arrowheads indicate the right boundaries of the nine promoter fragments. Sequence polymorphisms between the red- and non-red-skin promoters are boxed in blue and, from top to bottom, correspond to six SNPs, the 494-bp InDel, the 26-bp InDel, the 1-bp InDel, and the 2-bp InDel. The red box indicates the transcription start site (ATG codon).
